# Mental health and online learning among medical students during COVID-19 pandemic

**DOI:** 10.1192/j.eurpsy.2021.1744

**Published:** 2021-08-13

**Authors:** S. Aljhani, D. Alateeq, A. Alwabili, A. Alamro

**Affiliations:** 1 Department Of Psychiatry, Qassim University, Buraidah, Saudi Arabia; 2 Clinical Sciences, Princess Nourah bint Abdulrahman University, Riyadh, Saudi Arabia; 3 Medical Education, Qassim University, Buraidah, Saudi Arabia

**Keywords:** mental health, online learning, Medical Students, COVID-19

## Abstract

**Introduction:**

Medical students’ well-being is a concern that drawn interest.On March, 2020, WHO declared COVID-19 as a pandemic.Strict isolation measures and closing schools expected to influence the mental health of students.Online education introduced to adjust to new realities.These un-precedented circumstances create significant stress and challenges may lead to unfavorable effects on learning and the overall psychological health of students.

**Objectives:**

To explore the perception of stress and anxiety level among medical students in Saudi Arabia. To determine factors influencing perception of stress and anxiety among medical students in Saudi Arabia. To explore the association between perception of stress, anxiety and on-line learning.

**Methods:**

An online survey will be distributed through students representatives targeting medical students from different levels. The survey will have 4 components: 1) Demographic data that will include: age, gender, level of education and region of residence. 2) Questions concerned the experience of online learning. 3) Perceived stress scale by sheldon cohen. The perceived stress scale (PSS) is a 10-question tool will be used to measure a person’s perception of stress over the past month and scored as low, moderate and high perceived stress. 4)Generalized Anxiety Disorder 7 (GAD-7) which is also a valid tool in detecting anxiety in the last two weeks by 7 items with Likert scale answers which scored as minimal, mild, moderate and severe

**Results:**

Currently under analysis

**Conclusions:**

To be attached later

**Disclosure:**

No significant relationships.

